# Supplementary material for: Prevalence of Group II Introns in Phage Genomes
Source: bioRxiv. 2025 May 23:2025.05.22.655115. Preprint. [Version 1] doi: 10.1101/2025.05.22.655115 (PMC12139763; doi:10.1101/2025.05.22.655115)
Supplement: Supplement 1 [file media-1.pdf]

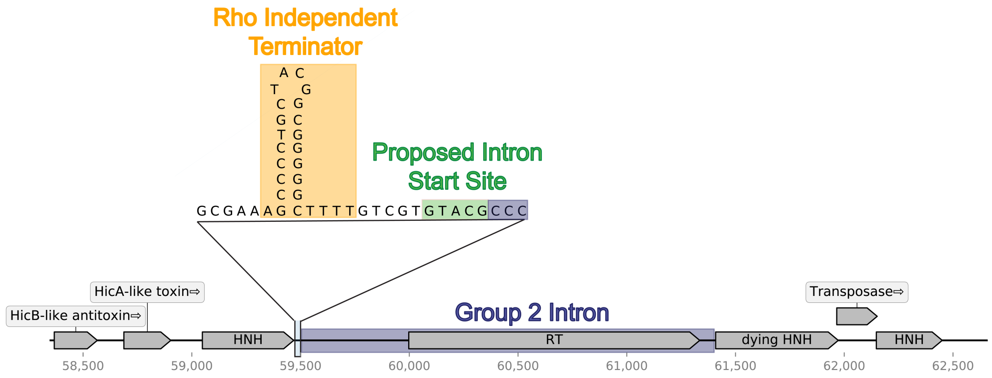


**Figure 1: A phage group IIC intron.** Group IIC introns typically insert 4-8 nucleotides downstream of a rho-independent transcriptional terminator, rather than in the coding region of their host gene. One of our phage group II introns is a type IIC intron, and we can identify a rho-independent terminator 5 nucleotides upstream of its insertion site.
